# Molecular Demonstration of a Pneumocystis Outbreak in Stem Cell Transplant Patients: Evidence for Transmission in the Daycare Center

**DOI:** 10.3389/fmicb.2017.00700

**Published:** 2017-04-24

**Authors:** Christine Robin, Alexandre Alanio, Maud Gits-Muselli, Giulia la Martire, Frédéric Schlemmer, Françoise Botterel, Cécile Angebault, Mathieu Leclerc, Florence Beckerich, Rabah Redjoul, Cécile Pautas, Andrea Toma, Sébastien Maury, Stéphane Bretagne, Catherine Cordonnier

**Affiliations:** ^1^Department of Hematology, Assistance Publique-Hôpitaux de Paris, Henri Mondor Teaching HospitalCréteil, France; ^2^Paris-Est Créteil UniversityCréteil, France; ^3^Parasitology-Mycology Laboratory, Assistance Publique-Hôpitaux de Paris, Lariboisière Saint-Louis Fernand Widal HospitalParis, France; ^4^Paris-Diderot, Sorbonne Paris Cité UniversityParis, France; ^5^Molecular Mycology Unit, National Reference Center of Invasive Mycosis and Antifungals, Centre National de la Recherche Scientifique, Institut Pasteur, URA3012Paris, France; ^6^Unit of Pneumology, Intensive Care Department, Assistance Publique-Hôpitaux de Paris, Henri Mondor Teaching Hospital, DHU A-TVBCréteil, France; ^7^Parasitology-Mycology Laboratory, Assistance Publique-Hôpitaux de Paris, Henri Mondor Teaching HospitalCréteil, France

**Keywords:** *Pneumocystis jirovecii*, genotyping, microsatellite, short tandem repeat, nosocomial infection

## Abstract

*Pneumocystis jirovecii* pneumonia (PCP) is a life-threatening infection in hematology. Although occasionally reported, the role of interhuman transmission of *P. jirovecii* in PCP, compared to that of reactivation, remains an unresolved question; the recommendation to isolate PCP patients in the hematology ward are not well evidence-based. Following an unexpected increase in the number of febrile pneumonia patients with *P. jirovecii* DNA detected in respiratory samples in our hematology ward, we explored 12 consecutive patients from November 2015 to May 2016. Genotyping of *P jirovecii* was performed using microsatellite markers. The frequency of simultaneous occupancy of these 12 patients in the same unit on the same day from 4 months prior to the first diagnosis was recorded. In three patients, the *P. jirovecii* genotype could not be determined because DNA was insufficient. One rare single genotype (Gt2) was found in four of the other nine, all allogeneic stem cell transplant recipients. The transmission map showed that these 4 patients had multiple opportunities to meet on the same day (median, 6.5; range, 4–10) at the daycare center. It was much less among the eight non-Gt2 patients (median, 1; range, 0–9; *P* = 0.048). This study, based on modern molecular technics, strongly suggests that interhuman transmission of *P. jirovecii* between allogeneic stem cell transplant recipients is possible. *P. jirovecii* DNA detected in respiratory specimens supports that isolation and respiratory precautions be recommended in such cases in the hematology ward.

## Introduction

*Pneumocystis jirovecii* pneumonia (PCP) is a life-threatening fungal complication in hematology patients, particularly recipients of allogeneic hematopoietic stem cell transplantation (HSCT), where mortality is greater than 50% (Cordonnier et al., 2016). It is usually accepted that *P. jirovecii* is acquired early in life and that individuals are continuously exposed to it through the air (Gajdusek, 1957; Gigliotti and Wright, 2012; Morris and Norris, 2012). However, it is unclear whether infection is the exclusive result of recent exposure or if reactivation of the early-acquired strain could also participate in the development of PCP. Person-to-person transmission has been documented during outbreaks, especially in solid organ transplant wards (Yiannakis and Boswell, 2016). To avoid such transmission, recent guidelines for managing PCP in hematology patients published by the European Conference on Infections in Leukemia group (Alanio et al., 2016c; Maertens et al., 2016; Maschmeyer et al., 2016) state that “it seems reasonable that severely immunocompromised patients should avoid contact with patients with documented PCP.” However, because data is lacking in this population, this is a C-III recommendation (e.g., there is poor evidence to support it). Similarly, the 2007 guidelines for isolation precautions from the US Centers for Disease Control and Prevention advises that PCP-infected patients should not be placed in the same room as immunocompromised patients, and other standard precautions should be taken (Siegel et al., 2007).

Genotyping studies of outbreaks in renal (Thomas and Limper, 2004; de Boer et al., 2011; Yiannakis and Boswell, 2016) or liver (Desoubeaux et al., 2016) transplant units were of major importance when reconsidering the mechanism of PCP development and supporting interhuman or environmental transmission of *P. jirovecii*. Additionally, the presence of *P. jirovecii* in the exhaled air of PCP patients (Choukri et al., 2010; Damiani et al., 2012) and the common genotype found in infected patients and colonized health care workers (Damiani et al., 2012) strongly suggest that PCP infection can also be nosocomial. However, only 16 of the reported outbreaks in kidney recipients thus far (Yiannakis and Boswell, 2016) were documented using molecular biology, and no firm statement on the transmission mode has been made.

The methods for detecting and genotyping *P. jirovecii* have greatly evolved over the last years. A PCP diagnosis increasingly relies on real-time quantitative PCR (qPCR) instead of microscopy and immunofluorescence staining (Alanio et al., 2016c). The qPCR assay increases the rate of detection and provides additional clues for elucidating the source of contamination. Various methods of genotyping have been developed (reviewed in Alanio et al., 2016c); multilocus sequence typing and internal transcribed spacer (ITS) sequencing are the most common. New genotyping methods based on short tandem repeat markers located in the nuclear genome are now available, allowing easier and more rapid analysis of clustered infections and accurate detection of mixtures (Parobek et al., 2014; Alanio et al., 2016b).

To our knowledge, this is the first possible interhuman transmission of *P. jirovecii* documented using modern molecular tools in the hematology ward. Microsatellite marker genotyping was used during an outbreak of febrile pneumonia in a hematology ward, and positive *P. jirovecii* detection was employed. This study shows that hematology patients can be infected by *P. jirovecii* genotypes transmitted from other at-risk patients in the ward. This clearly supports a strong recommendation for isolation and respiratory precautions where *P. jirovecii* is detected in respiratory DNA specimens from patients cared for in the same hematology ward.

## Materials and methods

### Study setting

The hematology department comprises 26 single-occupancy rooms for conventional hospitalization. It includes one unit (Unit 1) without air filtration that has eight beds, two units with laminar air-flow filtration that have six (Unit 2) and 12 (Unit 3) beds, and a daycare clinic, which cares for 30–50 patients daily on the upper floor. When the patients are at the daycare center, they all arrive between 8 and 9 a.m. and may stay several hours on site, sharing a unic waiting room. The department mainly recruits patients with acute leukemia, myelodysplastic syndrome, myeloproliferative disorders, and aplastic anemia. Around 40 allogeneic HSCTs are performed annually. Patients, hospital stays, and daycare center stays numbered 255, 505, and 2,450, respectively, and the median hospital stay was 20 days. These values have not appreciably varied since 2013.

Procedures for PCP prophylaxis in acute lymphoblastic leukemia and lymphoma patients and in HSCT recipients are in writing. The first-line recommended regimen is a single double-strength tablet of trimethoprim-sulfamethoxazole (TMP-SMX) thrice weekly, but if not tolerated, the second-line is atovaquone (750 mg oral suspension twice daily) or pentamidine aerosol monthly. Patients with pneumonia, except those in palliative care, are investigated by fiberoptic bronchoscopy and bronchoalveolar lavage (BAL) with a systematic search for *P.jirovecii* by May-Grünwald Giemsa (MGG) staining (RAL-555; RAL Reagents, Martillac, France) and an indirect immunofluorescence assay (IFA; MONOFLUO^TM^ kit, *P. jirovecii*; Bio-Rad, Marnes la Coquette, France) as previously described (Botterel et al., 2012). Since 2013, a qPCR targeting the mitochondrial LSU of the rRNA of *P. jirovecii* has been routinely performed in respiratory specimens (BAL fluid or upper respiratory tract samples) (Botterel et al., 2012). Other causes of pneumonia are investigated using systematic laboratory procedures to identify bacterial, viral, and other fungal pathogens as well as alveolar hemorrhage and alveolar proteinosis. A serum (1-3) β-D-glucan test (Fungitell assay, Associates of Cape Cod, Inc., Cap Cod, Massachusetts), when requested, was performed according to manufacturer recommendations and was considered positive at >80 pg/mL. However, the test was not routine at this time and could not be performed within 48 h of the BAL for all patients.

**Figure 1:**
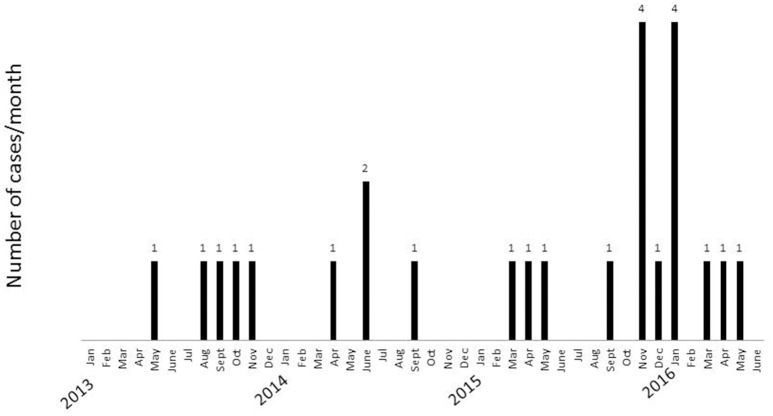
**Number of pneumocystis cases observed in the hematology department between January 2013 and May 2016**.

Figure 1 shows the frequency of febrile pneumonia and positive DNA tests for *P. jirovecii* between January 2013 and May 2016. It was 4 to 5 cases per year before the fall of 2015, but 4 cases were observed from November 6 to 14, 2015. Eight additional cases were observed from December 2015 to May 2016. This unexpected high incidence led to a search for evidence of transmission using *P. jirovecii* genotyping and an analysis of patient presence in the ward. According to French Health Public Law (CSP Art L1121-1.1), such an investigation does not require specific informed consent or ethics committee approval.

### Molecular typing

Molecular typing of *P. jirovecii* was performed on DNA extracted from respiratory specimen (BAL or/and sputum) that were frozen at −20°C. Microsatellite analysis was performed according to previously published methods (Gits-Muselli et al., 2015). Briefly, tests for six nuclear microsatellite markers (#022, #108, #138, #189, #278, and #279) were performed using fluorescent primers. The size of the PCR product obtained after capillary electrophoresis depends on the number of short tandem repeats for each marker. For a given marker, the size that is proportional to the number of repeats defines an allele. A unique combination of one specific allele per marker identifies a specific genotype. The genotypes were arbitrarily attributed numbers and were considered to be found if all alleles were unique for a given marker (pure genotype) or if more than one allele was found for one marker (multiple genotypes). Otherwise (i.e., when multiple alleles were observed for more than 1 marker), the genotype could not be determined, and the result was reported as a “mixture” of various genotypes.

## Results

### PCP patients

Twelve consecutive PCP patients hospitalized from November 2015 to May 2016 were included, and their characteristics are summarized in Table 1. All were investigated by fiberoptic bronchoscopy and BAL. Seven were allogeneic HSCT recipients. At BAL, all 12 patients were symptomatic, febrile, and hypoxemic, and lung CT images were consistent with interstitial pneumonia, mainly diffuse, patchy, ground-glass opacities. A serum (1-3) β-D-glucan test was available for nine patients and was positive for six (Patients 1, 3-6, and 10; Table 1). No serum (1-3) β-D-glucan test was performed for Patients 8, 9, and 12. At diagnosis, PCP prophylaxis was atovaquone in three patients and pentamidine aerosols in one because these patients were intolerant of TMP-SMX. Eight patients had no prophylaxis, five because of the absence of recommendations for mixed phenotype AL (*n* = 2) or for AML (*n* = 3) and one because of deep neutropenia after HSCT. In the other two, prophylaxis was stopped several months prior, after withdrawing immunosuppressive drugs. As a result, none of the 12 patients received TMP-SMX prophylactically.

**Table 1:** **Characteristics of the 12 patients who developed febrile pneumonia with positive detection of ***Pneumocystis jirovecii*** DNA in broncho-alveolar lavage fluid in the hematology ward between November 2015 and March 2016**.

**Patient No**.	**Age (years)**	**Sex**	**Underlying disease**	**Allogeneic HSCT before PCP, donor type**	**Date of diagnostic BAL**	**Microscopy results**	**qPCR result (Cq)**	***P. jirovecii* Gt^a^**	**Serum β-D glucane within ±48 h of BAL (value pg/mL)**	**PCP prophylaxis at time of PCP diagnosis**	**Possible concomitant causes of pneumonia**	**Outcome**
1	27	M	Myelodysplastic syndrome, secondary AML	No	Nov 6, 2015	MGG−; IFA−	37.2	Gt5	Positive (198)	None	*A. fumigatus* (positive culture on BAL fluid)	Treated, lost to follow-up 8 days after BAL
2	32	M	Hodgkin disease	Yes Unrelated donor	Nov. 9, 2015 (day 442 after HSCT)	MGG−; IFA−	34.6	Gt2 (pure)	Negative	Atovaquone (750 mgX2/d)	RSV (nasal swab and BAL) and *S. pneumoniae* (Blood cultures and BAL)	Treated, Recovered
3	49	F	Myelodysplastic syndrome	Yes One cord blood unit, no engraftment. 2^nd^haplo-identical HSCT	Nov 10, 2015 (day 90 after 1st HSCT)	MGG−; IFA−	40.0	Gt1 (pure)	Positive (93)	None	Alveolar hemorrhage	Treated, death from acute respiratory failure
4	51	M	ALL Ph+	Yes First HLA-identical HSCT in 2007. Relapse. Second HSCT from an unrelated donor	Nov 14, 2015 (day 304 after 2nd HSCT)	MGG+; IFA+	29.5	Mixture (including Gt3 alleles)	Positive (>500)	None	HSV in BAL	Treated, recovered
5	50	F	CLL	Yes Unrelated donor	Dec 21, 2015 (day 301 after HSCT)	MGG+; IFA+	26.7	Gt2 (pure)	Positive (>500)	Monthly aerosols of pentamidine	Rhinovirus on nasal swabs *A. fumigatus* in BAL	Treated, death from acute respiratory failure
6	68	F	Biphenotypic AL	No	Jan 9, 2016	MGG−; IFA−	35.6	Mixture	Positive (357)	None	–	Treated, recovered
7	65	M	ALL	Yes Unrelated donor	Jan 12, 2016 (day 418 after HSCT)	MGG−; IFA−	33.3	Gt2 (pure)	Negative	None	–	Treated, recovered
8	55	M	CLL	Yes HLA-identical donor	Jan 14, 2016 (day 543 after HSCT)	MGG−; IFA−	39.4	Not available (insufficient fungal DNA)	Nd	Atovaquone (750 mgx2/d)	–	Not treated. Switched to prophylactic TMP-SMX No recurrence
9	64	M	Biphenotypic AL	No	Jan 19, 2016	MGG−; IFA−	27.6	Gt3 + Gt4 (multiple)	Nd	None	–	Treated, recovered
10	65	M	Secondary myelofibrosis (thrombocythemia)	Yes HLA-identical donor	Mar 15, 2016 (day 250 after HSCT)	MGG+; IFA+	23.5	Gt2 (pure)	Positive (>500)	Atovaquone (750 mgx2/d)	–	Treated, death from acute respiratory failure
11	66	F	AML	No	April 15, 2016	MGG+; IFA−	32.8	Not available (insufficient fungal DNA)	Negative	None	–	Treated, recovered
12	26	F	AML salvage therapy	No	May 11, 2016	MGG−; IFA−	41.0	Not available (insufficient fungal DNA)	Nd	None	–	Not treated. Put on TMP-SMX prophylaxis. No recurrence

a *Those indicated as pure showed one allele per marker. Those indicated as multiple showed two alleles for one of six markers. Those indicated as mixture showed two alleles for more than one marker*.

*ALL, acute lymphoblastic leukemia; AML, acute myeloid leukemia; BAL, broncho-alveolar lavage; Gt, genotype; Cq, quantification cycle; CLL, chronic lymphocytic leukemia; HSCT, hematopoietic stem cell transplantation; IFA, immunofluorescence assay; MGG, May-Grünwald Giemsa (stain); Nd, not done; Ph+, chromosome Philadelphia positive; RSV, respiratory syncytial virus; TMP-SMX, trimethoprim-sulfamethoxazole*.

### Genotyping results

In three patients (Patients 8, 11, and 12), the *P. jirovecii* genotype could not be determined because insufficient amounts of DNA were obtained. In Patient 1, only one marker (#022) was amplified, but with a different allele from other patients, allowing the assumption that this patient had a specific genotype (Gt5). In the eight remaining patients, the six markers were correctly amplified. One genotype (Gt2) was found pure in four patients (Patients 2, 5, 7, and 10), all of whom were diagnosed within a timeframe of 4 months (November 9, 2015 to March 15, 2016). Of note, Gt2 had not been found in our collection of genotyped *P. jirovecii* sspecimens from France and Europe (*n* > 300 in total) (Alanio et al., 2014, 2016a; Gits-Muselli et al., 2015). The various Gt2 alleles were searched for but not found in the other patients, confirming that they did not harbor it.

Patients 4 and 9 shared Gt3 genotype, in addition to other alleles in Patient 4, and in addition to Gt4 in Patient 9. Of note, Gt3 has been very rarely observed in our experience (one patient from Paris, one from Germany, and one from The Netherlands out of at least 300 patients).

### Transmission map

**Figure 2:**
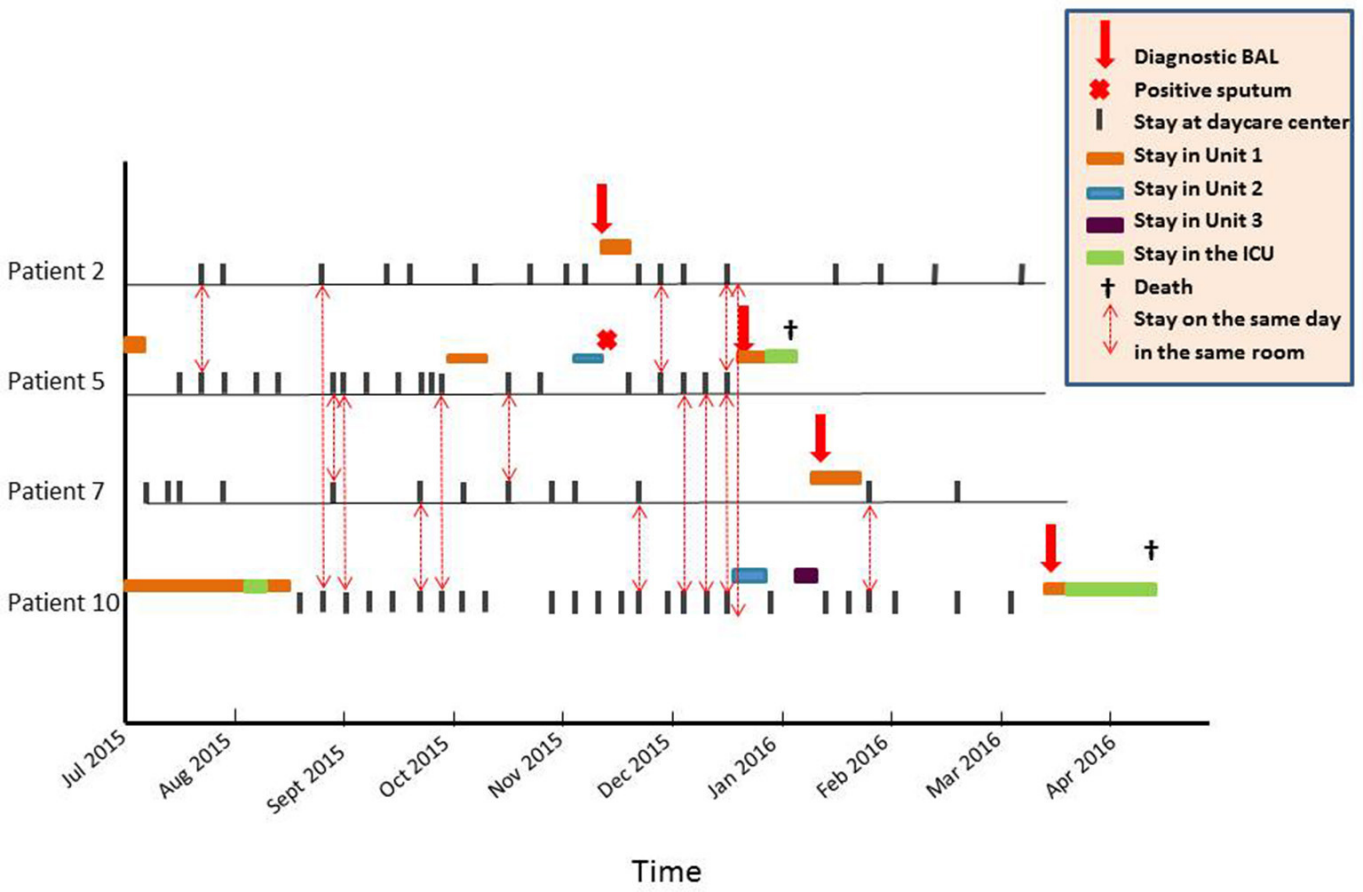
**Transmission map of the 4 patients infected with genotype 2 ***Pneumocystis jirovecii*** in the hematology ward, showing that any meeting between two Gt2 patients must have been in the daycare center because no 2 patients were concomitantly hospitalized in the same unit of the ward**. Patient 2 met Patients 5 and 10 once each before developing PCP and twice each after developing PCP while under treatment followed by secondary prophylaxis, including once when both were met on the same day. Patient 2 never met Patient 7. Patient 5, the only one to have met all 3 others at least once, met Patient 2 three times, Patient 7 twice, and Patient 10 five times. Patient 7 met Patient 5 twice and Patient 10 three times (twice before the diagnosis of PCP, and once thereafter). Patient 7 never met Patient 2. Patient 10 met Patient 2 twice and Patient 5 five times, including one occurrence where both were met on the same day. Patient 10 met Patient 7 three times.

Gt2 was first observed on November 9, 2015 in the BAL of Patient 2 (Figure 2). To explore a possible nosocomial transmission through close contact between the four Gt2 patients, we collected all the dates, times of stays, and meeting sites within the hospital from July 2015 to May 2016 (i.e., starting 4 months before and ending 6 months after the first case). Direct transmission was deemed possible if patients were found to be in the same room on the same day. All contacts between these four patients occurred in the daycare center, which has only one waiting room (Figure 2). The median number of contacts was 6.5 (range, 4–10). In contrast, the median number of contacts between the other eight patients and any Gt2 patient was significantly lower (median, 1; range, 0–9; *P* = 0.048). Additionally, non-Gt2 to non-Gt2 patient contact was zero for five patients, one for one of them, and three for two of them. Figure 2 strongly suggest that either patient 2 or patient 5 was the source of Gt2 transmission.

Moreover, 1 month before diagnosis in December 2015, Patient 5 was hospitalized for fever, bronchopulmonary symptoms, and hypoxia in a different unit than Patient 2. The chest CT scan showed micronodules in the upper right lobe, indirect investigations were negative, and the patient refused fibroscopy. *P. jirovecii* was found via PCR in sputum collected on November 10, but the result of the test was missed. Improvement occurred after taking antibacterials, and prophylactic aerosols of pentamidine were reinstated, but 4 weeks later, the patient was hospitalized for febrile dyspnea. A BAL documented full-blown PCP with positive microscopy and PCR. She died from respiratory failure. We retrospectively determined from a sputum sample that the *P. jirovecii* genotype was pure Gt2.

Although Patients 4 and 9 share the rare Gt3 genotype, they never met.

## Discussion

We report here the documentation of interhuman transmission of *P. jirovecii*, which was between 4 allogeneic HSCT recipients. We used *P. jirovecii* genotyping and analysis of the transmission path in the hospital, which revealed that transmission probably occurred in the daycare center (Figure 2) where the patients repeatedly met for post-transplant follow-up. Contacts between the four Gt2-patients and the concomitance of PCP infection in Patient 2 and *P. jirovecii* detection in Patient 5 make it plausible that either Patient 2 or Patient 5 could have been the first transmitter. The Gt2 transmission could have started from the first PCP case (Patient 2), who transmitted the disease to Patients 5 and 10, either of whom could have contaminated Patient 7. Alternatively, because Patient 5 was retrospectively identified as a Gt2 carrier, she could have been the first transmitter.

This transmission likely occurred because of the absence of effective PCP prophylaxis. Indeed, out of 30 PCP outbreaks reviewed by Yiannakis et al., in 2016, 25 (83%) were reported in kidney transplant recipients, and most were not receiving PCP prophylaxis (Yiannakis and Boswell, 2016). In the hematology ward, there are usually written procedures for PCP prophylaxis, at least for the most at-risk patients (i.e., allogeneic HSCT recipients, acute lymphoblastic leukemia and lymphoma patients). The first-choice prophylactic regimen in at-risk patients is TMP-SMX (Stern et al., 2014; Maertens et al., 2016). However, this can be associated with side effects, poor compliance, or omission. Although the staff complies with our policies, none of the 12 patients included in this study was receiving TMP-SMX at the time of PCP diagnosis. Because all four Gt2 patients were allogeneic HSCT recipients, they might have been at higher risk because of their repetitive, often weekly, appointments at the daycare center in spite of being a minority of our study population.

Until now, only two PCP outbreaks have been reported in hematology wards. In one, five diagnoses were reported within 6 months in the same unit but without molecular investigation (Ong and Jones, 1998). In the other, nested amplification, cloning, and sequencing of ITS showed a common ITS sequence type between two of the eight hematology study patients who shared the same room (Helweg-Larsen et al., 1998). However, the genotype was also the most common found in Denmark, where the study was performed. Therefore, the authors concluded that “person-to-person transmission may occur but it may be a relatively infrequent event” (Helweg-Larsen et al., 1998). On the other hand, a 2013 Belgian survey including 21 patients with *P. jirovecii* based on PCR of BAL fluid in the same hospital with six hematology patients failed to find transmission of the same *P. jirovecii* genotype using multilocus sequence typing (Depypere et al., 2016). In our series, nosocomial transmission in the daycare center between four of the 12 patients is amply supported. First, these patients shared a unique *P. jirovecii* genotype that had never been observed in our experience on more than 300 *P. jirovecii* strains across Europe (Gits-Muselli et al., 2015; Alanio et al., 2016a) while 70–92% of PCP cases harbor mixtures of genotypes (Gits-Muselli et al., 2015; Alanio et al., 2016b; Depypere et al., 2016). Second, they had multiple opportunities to meet in the daycare center where they repetitively stayed several hours in the same, confined, waiting room. Finally, all were deeply immunosuppressed, and three were receiving no PCP prophylaxis.

Sharing the same genotype does not allow determination of the index case. Indeed, we do not know when transmission eventually occurred, but because a single pure genotype (Gt2) was found in four patients, a recent acquisition in these patients is suggested. Whether the transmission of Gt2 was direct, interhuman, the result of close contact, or was indirect through the environment can also not be determined. Healthcare workers could be the culprits, as suggested in other studies (Vargas et al., 2000; Miller et al., 2001; Valade et al., 2015), but this was not explored. Similarly, 16 outbreaks in kidney transplant patients have been documented using various molecular methods, and a single or predominant strain was found in 13 of them, supporting interhuman transmission either from patient to patient, via healthcare workers, or through the environment (Yiannakis and Boswell, 2016). However, in none of these reports could all cases be ascribed to the same genotype, suggesting that both mechanisms of infection—reactivation and recent interhuman transmission—probably occurred concomitantly, whether or not in the setting of an outbreak. Multiple *P. jirovecii* genotypes could circulate at the same time, and from various people. As genotype mixtures are common in PCP patients (Gits-Muselli et al., 2015; Alanio et al., 2016b; Depypere et al., 2016), this precludes any conclusion about which genotype was initially present. Only monitoring of patients over a long period of time could elucidate this fact.

While it is important to note that transmission occurred between our study patients, one may consider that PCP was not confirmed in five of those included in this study. Indeed, Patients 2, 7, 8, 9, and 12 were microscopically negative using MGG or IFA staining and were serum β-D-glucan negative. However, all patients had clinical and imaging patterns consistent with PCP. Even if some patients are considered probable or putative cases, it does not change the possibility of *P jirovecii* transmission among them. We do not know whether there is a minimal fungal load to contaminate another patient. It could be hypothesized that any cough generated by another bronchopulmonary infection, especially a respiratory viral infection, could contribute to increased transmission of *P. jirovecii*, whatever the fungal load. Therefore, our study confirms the interest of adding a *P. jirovecii* qPCR on respiratory samples of hematology patients with pulmonary symptoms to determine whether they are possible transmitters (Guigue et al., 2015).

To determine whether patients with low fungal loads are the source of infection or participate in the transmission chain is hampered by the inability to genotype when facing a low fungal burden. The qPCR used for *P. jirovecii* detection in respiratory samples targets a multicopy gene (mtLSUrRNA) to improve test sensitivity (Alanio et al., 2011; Botterel et al., 2012), whereas the alleles tested for genotyping are located on single-copy genes (Gits-Muselli et al., 2015). Therefore, other epidemiological links or transmission between patients harboring low fungal loads could have been missed. A typing system based on the investigation of mitochondrial markers (mainly by sequencing mtLSU or cytb genes) for genotyping would lead to increased sensitivity, but the reliability of this method is disputable because mitochondrial heteroplasmy and recombination could occur (Valero et al., 2016). Furthermore, the plasticity and mutation rates is known to differ between the mitochondrial genome and the nuclear genome. This is why mitochondrial genes have not been retained in the multilocus sequence typing approach for *Candida albicans*, for instance (Odds and Jacobsen, 2008).

In conclusion, this study illustrates two important features: (1) nosocomial transmission of *P. jirovecii* is possible between infected patients within the hematology ward, as illustrated in our daycare center; and (2) TMP-SMX, the optimal PCP prophylaxis (Maertens et al., 2016) is important, and its absence increases the susceptibility of at-risk patients during an outbreak. This clearly supports a strong, specific recommendation for isolation and respiratory precautions with PCP, and more generally, encourages reinforcing control policies when pulmonary symptoms are seen in the hematology ward. This recommendation was, up to now, not evidence-based for PCP in hematology (Siegel et al., 2007; Maertens et al., 2016). Thus, hospitalized PCP patients should be in single-occupancy rooms and wear at least a surgical mask to prevent transmission when meeting other patients in confined waiting rooms, such as a daycare center. Wearing a mask could have prevented the onset of this outbreak. Our data support that these recommendations be extended to any PCP-at-risk patient with febrile pneumonia and the presence of *P. jirovecii*, detected using qPCR on respiratory specimens. Wearing a mask in the hospital for any hematology patient without respiratory symptom should logically decrease the transmission of any pathogen transmitted through air, and subsequently decrease the incidence of other respiratory diseases, especially during the fall-winter season. The efficacy and acceptability of such measure should, however, be carefully assessed.

## Author contributions

CR, AA, and CC conceived and designed the study. CR, AA, MG, GL, FS, ML, FlB, RR, CP, AT, and SM provided study materials or patients. CR, AA, GL, FrB, and CA collected and assembled data. CR, AA, CC, and SB analyzed and interpreted data. CR, AA, SB, and CC drafted the manuscript. All authors approved the final version.

### Conflict of interest statement

The authors declare that the research was conducted in the absence of any commercial or financial relationships that could be construed as a potential conflict of interest.
